# Testing therapeutics in cell-based assays: Factors that influence the apparent potency of drugs

**DOI:** 10.1371/journal.pone.0194880

**Published:** 2018-03-22

**Authors:** Elena Postnikova, Yu Cong, Lisa Evans DeWald, Julie Dyall, Shuiqing Yu, Brit J. Hart, Huanying Zhou, Robin Gross, James Logue, Yingyun Cai, Nicole Deiuliis, Julia Michelotti, Anna N. Honko, Richard S. Bennett, Michael R. Holbrook, Gene G. Olinger, Lisa E. Hensley, Peter B. Jahrling

**Affiliations:** 1 Integrated Research Facility, Division of Clinical Research, National Institute of Allergy and Infectious Diseases, National Institutes of Health, Frederick, Maryland, United States of America; 2 Emerging Viral Pathogens Section, National Institute of Allergy and Infectious Diseases, National Institutes of Health, Frederick, Maryland, United States of America; University of Texas Medical Branch at Galveston, UNITED STATES

## Abstract

Identifying effective antivirals for treating Ebola virus disease (EVD) and minimizing transmission of such disease is critical. A variety of cell-based assays have been developed for evaluating compounds for activity against Ebola virus. However, very few reports discuss the variable assay conditions that can affect the results obtained from these drug screens. Here, we describe variable conditions tested during the development of our cell-based drug screen assays designed to identify compounds with anti-Ebola virus activity using established cell lines and human primary cells. The effect of multiple assay readouts and variable assay conditions, including virus input, time of infection, and the cell passage number, were compared, and the impact on the effective concentration for 50% and/ or 90% inhibition (EC_50_, EC_90_) was evaluated using the FDA-approved compound, toremifene citrate. In these studies, we show that altering cell-based assay conditions can have an impact on apparent drug potency as measured by the EC_50_. These results further support the importance of developing standard operating procedures for generating reliable and reproducible *in vitro* data sets for potential antivirals.

## Introduction

Ebola virus (EBOV) infection in humans and nonhuman primates is often associated with high morbidity and mortality rates, as well as severe hemorrhagic fever [1–4]. EBOV is a biosafety level–4 pathogen transmitted by contact with bodily fluids, fomites, or droplets from infected patients. EBOV is considered a significant threat to public health and global security due to its potential to be used as a bioweapon [5–8]. Currently, no FDA-approved vaccine or therapeutic agents are available, and supportive care remains the standard for Ebola virus disease (EVD) treatment. Therefore, accelerated efforts in the development of therapeutics is a key objective in the EBOV research community, especially since the 2013–2016 EVD epidemic in Western Africa.

Drug discovery and development requires considerable time and resources to identify an effective drug that will progress to clinical trials [9, 10]. As a result, research investigating the repurposing of drugs for additional indications have become increasingly more prevalent to accelerate the identification of therapeutic drugs for EVD. The off-label use of FDA-approved drugs is particularly advantageous as safety concerns and ethical problems have already been addressed [11–14].

To effectively identify potential compounds of interest from large libraries of chemical compounds, share more reliable and reproducible data between laboratories, and provide data to the international community, appropriate methods or models need to be established. Furthermore, these models should be evaluated to determine how predictive they are for identifying compounds most likely to be efficacious in humans. For EVD, indications of efficacy could include successful treatment and survival of patients, alleviation of disease severity, or mitigation of clinical symptoms associated with EBOV infection.

A variety of methods are available to measure antiviral activity *in vitro*. However, the development of a screening assay to detect compounds with anti-EBOV activity was previously limited due to the difficulty of developing a suitable high-throughput screening system for a biosafety level-4 viral pathogen. Classical methods evaluate drug efficacy include the reduction of virus yield [15] or a decrease in viral RNA transcription as determined by real-time polymerase chain reaction (PCR) [16–18]. Recent therapeutic screening methods have transitioned from the classical methods of measuring viral inhibition to assays with the ability to be automated, resulting in higher-throughput. Assay chemistries have been developed to enable the homogeneous measurement of a variety of different endpoints such as cytopathic effect, viral protein or reporter gene expression, which can serve as markers of viral replication [19]. The growing interest in identifying drugs with activity against EBOV has resulted in a variety of assays and readouts for activity as well as cytotoxicity. The use of cell-based assays for high-throughput screening of compound libraries has increased steadily over recent years [20–23]. As cell-based assays monitor specific viral proteins and provide the means to screen for potent viral inhibitors intracellularly, these assays identify drug candidates with desired pharmacological properties in the primary drug-discovery pipeline.

In this study, the susceptibility to EBOV using both immortalized cell lines and primary monocyte-derived macrophages (MDMs) was investigated under a variety of conditions such as different multiplicities of infection (MOIs), times of exposure, and the cell passage numbers. A cell-based assay with EBOV VP40-specific antibody was used to detect infected cells. Fluorescence or chemiluminescence readout was used for determining signal-to-noise (S/N) ratio, and a high-content imaging system was applied to determine the percentage of EBOV-positive cells. Toremifene citrate, which the World Health Organization considered evaluating in a clinical trial for treatment of EVD, was chosen as a positive control to measure EBOV inhibition under each condition [24–27].

The conditions under which drugs are tested can influence their apparent potency. While testing drugs for the World Health Organization (WHO) community, we received many requests to repeat experiments under specific conditions to confirm activity identified by another laboratory. The resulting data sets indicated just how variable the EC_50_ value can be under varying assay conditions. The data presented here provides insight on how different assay parameters can impact the *in vitro* efficacy of potential anti-EBOV antivirals using toremifene citrate as a model compound.

## Materials and methods

### Cells and compounds

Vero E6 (African green monkey kidney; ATCC 1586) cells were obtained from the American Type Culture Collection (Manassas, VA). Vero C1008 (E6) cells (African green monkey kidney, working cell bank NR-596) were obtained through BEI Resources (National Institute of Allergy and Infectious diseases [NIAID], National Institutes of Health [NIH], Manassas, VA). Huh 7 cells (human hepatocellular carcinoma) were obtained from Dr. Hideki Ebihara (NIAID, Rocky Mountain Laboratories, Hamilton, MT). All cell lines were maintained at the Integrated Research Facility (IRF) following cell source instructions. A primary Vero E6 and Huh 7 cells culture were grown to 90% confluency in a T-175 (Fisher Scientific) or triple layer tissue culture flask (Nunc) containing Dulbecco’s modification of Eagle medium (DMEM) (Gibco) supplemented with 10% heat-inactivated fetal bovine serum (FBS) (Sigma). Cells were dispersed by trypsin (Gibco) treatment and then reseeded into secondary cultures. The process of removing cells from the primary culture, diluting, and then transferring them to secondary cultures constitutes a passage. Both cell lines were provided at passages 4–22, at which point a new culture was introduced and the previous passage series was ended. Additionally, cell cultures were required to be a least 85% viable in order to achieve acceptance criteria and to be plated for use in a screening assay.

The generation of MDMs has been described in previous studies [28, 29]. Briefly, PBMCs were isolated from human whole blood by density-gradient centrifugation over Histopaque (1.077 g/ml, Sigma-Aldrich, St. Louis. MO). Monocytes were purified using human CD14-specific microbeads (Miltenyi Biotec, San Diego, CA, 130-050-201) following manufacturer’s instructions. CD14^+^ monocytes were differentiated into MDMs by culturing for 6–7 days with recombinant human macrophage colony-stimulating factor (Bio-Techne, Minneapolis, MN, 216-MC-005) and conditioned medium from KPB-M15 cells (kind gift from Dr. Atsunobu Hiraoka, SCGF Research Laboratory, Kyoto, JP). Media were replaced every 2–3 days during the incubation for a total of 6–7 days. The cells were harvested and plated on desired 96-well plates 1 day prior to the drug screen assay. The differentiated MDMs were characterized by flow cytometry before assay initiation. Toremifene citrate (oral solution) tested in this study was purchased from Sigma-Aldrich (CAS 89778-27-8; T7204-5MG).

### Virus isolation

The Makona 05 isolate of EBOV (H. sapiens-tc/GIN/14/WPG-C05) (EBOV/Mak, GenBank accession no. KP096420), a kind gift from Dr. Gary P. Kobinger (Public Health Agency of Canada, Winnipeg, CA), was used in these studies. To generate virus stocks, EBOV/Mak was inoculated at an MOI of 0.01 in Vero C1008 cells (BEI resources, Manassas, VA, catalog NR-596). When the cytopathic effect was visible at day 5–7 after infection (51–75% of monolayer showing CPE), cell culture supernatants were harvested and clarified by centrifugation. The EBOV/Mak titer was determined by plaque assay in Vero E6 cells. Virus titers were measured using 10-fold serial dilutions of culture supernatant in triplicate infections of Vero E6 cell monolayers in 6-well plates. After incubation at 37°C for 1 h (plates were rocked every 15 minutes), 2 ml of medium containing 2X MEM (Gibco), 1.25% Avicel (FMC Biopolymer), and 1X Antibiotic-Antimycotic (Gibco) were added to each well (2ml/well). After 7 days post-incubation, virus plaques were stained with 0.2% crystal violet (Ricca Chemical) in 10% Neutral Buffered Formalin (Thermo Scientific) and infectivity titers were measured in plaque forming units per ml (PFU/ml). All procedures using live EBOV were performed under biosafety level-4 (BSL-4) conditions.

### Immunofluorescent staining and imaging (without drug addition)

Vero E6 and Huh 7 cells were seeded overnight at 3 to 4 × 10^4^ cells per well and MDM cells were plated at 1 × 10^5^ cells per well in 100 μl of Dulbecco’s modified Eagles’s medium with 10% fetal bovine serum in black opaque (Thermo Fisher Scientific, Waltham, MA, Corning 3916, 07-200-627) or clear bottom 96-well Greiner microplates (Greiner Bio-One, Monroe, NC, 655948). EBOV/Mak isolate was diluted in culture media to the specified MOIs (the titers used to determine MOI hereby were generated on Vero E6 cells) in 96-well plates. The cells were then infected by transferring 50 μl of EBOV/Mak isolate from the virus dilution plates to cell plates using the 96-well liquidator (Rainin Instrument, Oakland, CA). The cells were incubated at 37°C and 5% CO_2_ for the indicated periods of time. The plates were fixed by adding 200 μl of 20% neutral-buffered formalin (final concentration 10%) at 24, 48, 72 or 96 h post-inoculation (hpi). After fixing for 24 h, the plates were transferred to a BSL-2 lab for antibody staining as described previously [28]. Briefly, EBOV was detected by exposure of the infected, fixed, and permeabilized cells to a monoclonal mouse antibody specific to the EBOV VP40 matrix protein (B-MD04-BD07-AE11, made by US Army Medical Research Institute of Infectious Diseases, Frederick MD under Centers for Disease Control and Prevention contract) [30], followed by staining with an AlexaFluor^®^ 594 goat anti-mouse IgG (heavy + light chains) antibody (Life Technologies, Carlsbad, CA) at 37°C for 1 h. Fluorescence was quantified using a Tecan plate reader (Infinite^®^ M1000, Tecan US, Morrisville, NC) or an high-content imaging (HCI) system (Operetta, PerkinElmer, Waltham, MA). HCI images were collected at 20x magnification using 1 to 9 fields of view in each well to quantify the percent of EBOV-positive cells. The viability of the cell layer was monitored by staining cell nuclei with the Hoechst 33342 dye (Molecular Probes) at 37°C for 2 h. Columbus 2.4.2 software (PerkinElmer) was used to analyze the HCI data.

The chemiluminescent enzyme-linked immunosorbent assay (CELIA) was performed by detecting EBOV with the anti-EBOV VP40 antibody followed by staining with the horseradish peroxidase (HRP)-conjugated goat anti-mouse secondary antibody (SeraCare, Milford, MA, cat. #074–1802). Chemiluminescence was quantified using Pico Chemiluminescent Substrate (Thermo Fisher Scientific Inc., Rockford, IL) and a plate reader (Infinite^®^ M1000 Tecan).

### Drug screening by cell-based assay and cytotoxicity testing

**Fig 1:**
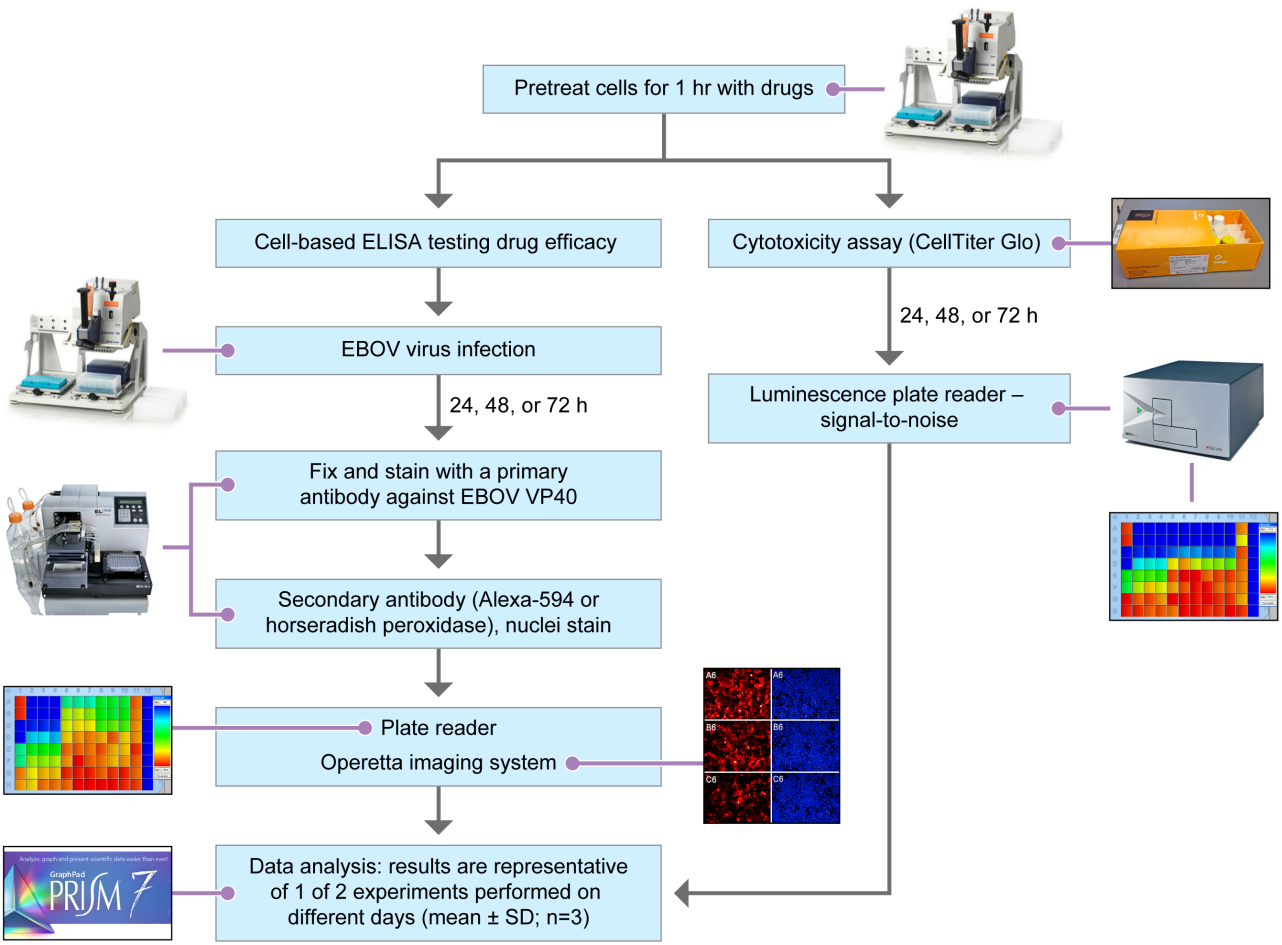
Flow chart of the steps of the EBOV drug screen assay. Cells and media are prepared in 100 μl/well cell plates and incubated overnight. Drugs in 50 μl/well are transferred from drug dilution plates to cell plates using a 96-well manual benchtop pipettor for 1 h of contact. In the biosafety level-4 (BSL4) laboratory, EBOV in 50 μl/ well is transferred to the cell/drug plates using a 96-well manual benchtop pipettor for a final volume of 200 μl/well. At specific assay endpoints, cells are fixed and transferred to the BSL-2. Immunostaining was performed with a EBOV-specific antibody against VP40 and a fluorescent or chemiluminescent secondary antibody using a plate washer/Dispenser. Fluorescence is quantified on a plate reader. The HCI system (Operetta) is used to detect EBOV-positive cells and count cells with a nuclei stain (Hoechst 33342). In parallel, cytotoxicity assays (CellTiter Glo) with mock infected cells are performed at BSL-2. Luminescence is read on the Infinite^®^ M1000 Tecan plate reader. Data are analyzed using GraphPad Prism and/or Columbus software (Operetta).

Vero E6, Huh 7 and MDM cells were seeded as described above in 100 μl media overnight at 37°C with 5% CO_2_. Compounds in dimethyl sulfoxide were prediluted to reduce dimethyl sulfoxide concentration to 0.05% or lower. Compounds were prediluted in dilution blocks before performing a final 1:4 dilution by transferring 50 μL of each compound to cell plates containing 150 μL of cell culture media. This dilution achieved a desired compound concentration in 200 μl of cell culture media. The process of performing the drug screen assay is shown in Fig 1. Three plates were set up per experiment, two plates (clear bottom 96-well Greiner microplates) for detecting inhibition of EBOV, and one mock plate (black opaque plate) for determining drug cytotoxicity. After 1 h of predilution and transport to the BSL4 laboratory, 50 μl of virus (or mock control) at the desired MOI was added to cells. At 48, 72 or 96 hpi, assay plates were fixed at final concentration of 10% NBF for 24 h before transferring to a BSL-2 lab for staining. Infected cells were detected as described above. To further confirm the accuracy of assays with high background, chemiluminescence assay was performed afterwards.

Cytotoxicity in mock infected cell plates was measured 48 or 72 h after treatment with compounds using the CellTiter Glo luminescent cell viability assay kit according to the manufacturer’s instructions (Promega, Madison, WI). Luminescence was read on the Infinite^®^ M1000 Tecan plate reader (Fig 1).

### Statistical analysis

Non-linear regression analysis and curve fitting parameter were performed to calculate EC_50_s, EC_90_s and 50% cytotoxic concentration (CC_50_s) (GraphPad Software, La Jolla CA) [12] using dose-response curves for the compounds (toremifene citrate). Error bars of dose-response curves represent the standard deviation of three replicates. Equations for the ratio of S/N, percentage of EBOV-positive infected cell, and Z' factor were defined previously [31, 32], and EC_50_s were used as parameters for assay validation. The quality control of cell-based assay is represented by Z' factor which is defined as equation Z' = [1-((3*SD_pos_)+(3*SD_neg_))/(Imean_pos_-Imean_neg_)]. In our assay, the Z' criteria is as follows: Z' = 0.5–1.0 corresponds to an excellentassay; Z' = 0–0.5 corresponds to a suboptimal assay; Z' <0 corresponds to a unsuccessful assay [32].

## Results

### Comparison of EBOV replication in three different cell types

The susceptibility to EBOV infection was evaluated in multiple cell types in cell-based assays measuring anti-EBOV activity. Vero E6, Huh 7 and MDM cells were infected with EBOV/Mak isolate at 8 different MOIs, and the assay was terminated after 24, 48, 72 or 96 hpi. Cells were stained with a fluorescent antibody, and Hoechst dye was used to visualize the cell nuclei. Infectivity was measured using the high-content imaging (HCI) system as percentage of VP40 positive cells. The growth of EBOV/Mak isolate in three cell types was compared over time.

**Fig 2:**
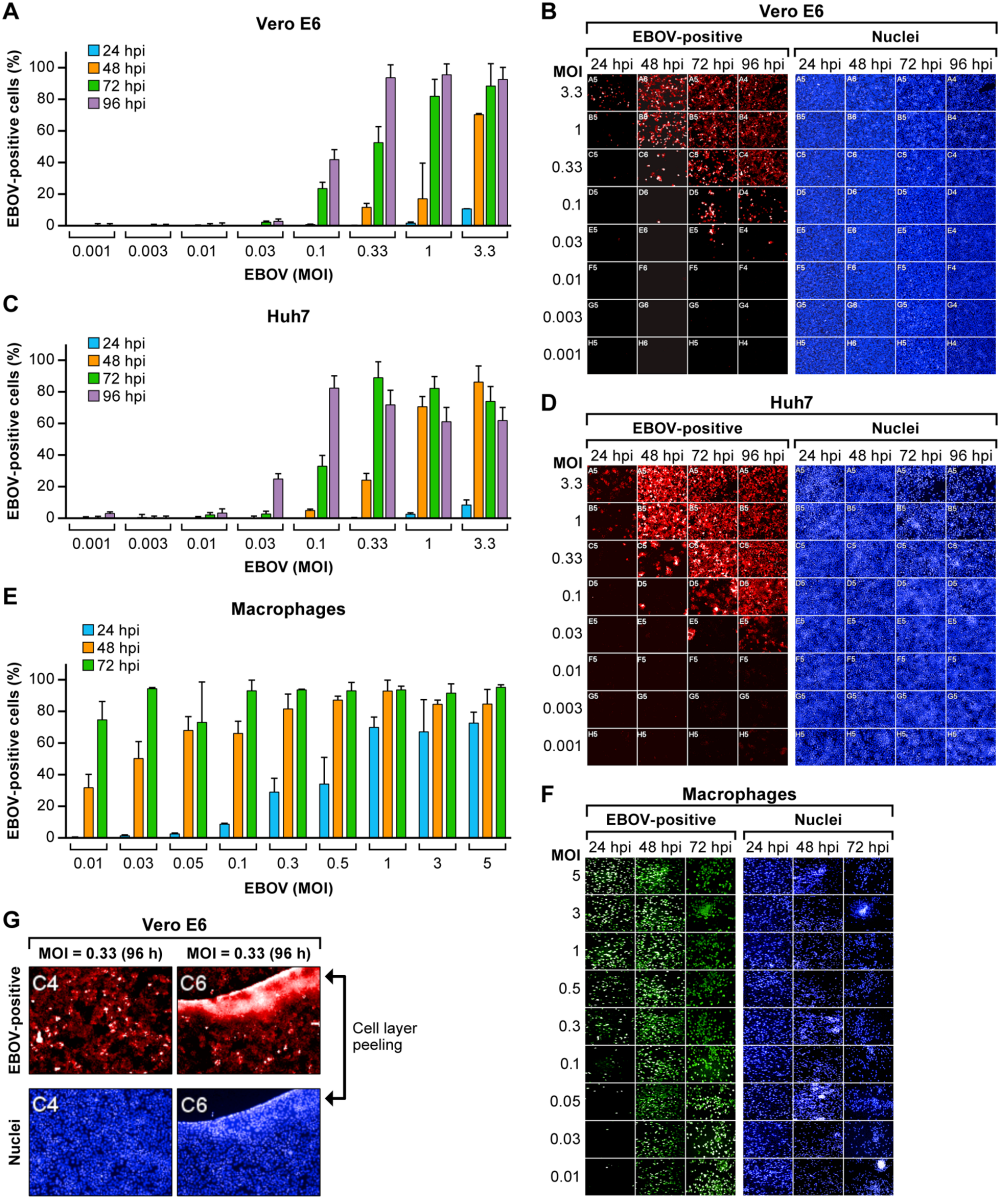
Susceptibility of different cell types to infection with EBOV. Vero E6 (A, B), Huh 7 (C, D), and MDM (E, F) cells were infected with EBOV/Mak at a range of MOIs. The assay endpoints were 24, 48, 72 or 96 hpi. Following staining with a VP40-antibody, HCI was performed to determine the percentage of EBOV-positive cells. Corresponding HCI images for EBOV-positive cells and nuclei (B, D, F) are shown. (G) Cell layer viability. Vero E6 cells were infected with EBOV/Mak at an MOI of 0.33 for 96 h. Two representative well images are shown; one image (well C4) has an intact cell layer, while the other image (well C6) shows the cell layer peeling off the well surface. Top panels show cells stained with the anti-EBOV VP40 antibody, bottom panels show the nuclei stain. Values are the average of triplicate wells (mean ± SD; n = 3). Abbreviations: EBOV, Ebola virus; hpi, hours post-inoculation; HCI, high-content imaging; Mak, Makona; MOI, multiplicity of infection; SD, standard deviation; VP40, EBOV VP40 matrix protein.

In Vero E6 cells, EBOV spread slightly slower, and the number of positive cells were overall lower compared to Huh 7 cells (Fig 2A–2D). MDMs were the most susceptible to EBOV among the cell types. At 24 hpi, MDMs already exhibited a typical dose response relative to virus input, while only minimal EBOV replication was observed in Vero E6 and Huh 7 cells at 24 hpi at all MOIs tested. Virus spread effectively at 48 hpi with higher (1 to 3.3) MOI of Vero E6, Huh 7 cells and most of MOIs in MDMs (Fig 2A–2F). The infection in MDMs was saturated at 72 hpi at almost all MOIs (Fig 2E and 2F). The nuclear stain for all cell types showed that the cell layer deteriorated with increased virus inoculum and duration of infection as was clearly evident at the 96 hpi time point and at higher virus input (MOIs of 3.3) (Fig 2B, 2D, 2F and 2G). The cell layer frequently became fragile, and at later time points, the cell layer would lift off the well surface possibly because of increased exposure to virus, higher MOI, or manipulation of plates during the fixing/staining procedure (Fig 2G). The HCI system proved to be invaluable for determining optimal assay conditions (end point and virus input) and in identifying potential issues such as cell layer integrity.

### Comparison of fluorescence-based detection methods

Different virus input and endpoints were assessed to identify an acceptable range for testing drugs in Vero E6 and Huh 7 cells (Tables 1 and 2). The same experimental plates from Fig 2A–2D were used for this analysis. The fluorescence S/N ratio was calculated using a Tecan plate reader, and the percentage of EBOV-positive cells was determined using HCI. For reproducible data, we determined that the percentage of EBOV-positive cells should be kept within a range of 10 to 80% and the S/N ratio at 4 to 10. At lower S/N ratios (<4) or lower percentage of infected cells (<10%) distinguishing activity of a drug from non-activity becomes increasingly difficult. On the other hand, at higher S/N ratios (>10), when cell infection rate trends towards 80–90% and cells overgrow at later time points, the risk of a disrupted cell layer is greater. Cell layer disruption increases the variability of the assay. Therefore, the optimization of assay conditions (i.e., duration of infection, virus input) was carefully calibrated resulting in a compromise between signal strength and cell viability.

**Table 1:** Effect of time postinoculation and virus inoculum on the fluorescent EBOV drug screen assay on Vero E6 cells.

MOI^a^	24 hpi^b^		48 hpi		72 hpi		96 hpi	
	EBOV^+^ (%)^c^	S/N^d^	EBOV^+^ (%)	S/N	EBOV^+^ (%)	S/N	EBOV+ (%)	S/N
**0.0010**	0.0	0.5	0.0	1.0	0.0	1.4	0.1	1.3
**0.0033**	0.0	0.8	0.0	1.1	0.0	1.0	0.0	0.9
**0.01**	0.0	0.9	0.2	1.1	0.0	1.4	0.3	1.5
**0.03**	0.1	1.1	0.0	0.6	2.3	0.8	2.8	1.5
**0.10**	0.0	1.1	0.9	1.1	23.6	3.9	41.8	6.4
**0.33**	0.3	1.0	11.7	2.7	52.5	10.2	93.7	8.2
**1.00**	1.8	1.5	17.1	3.8	81.9	10.7	95.5	6.9
**3.33**	10.6	3.1	70.3	9.1	88.4	14.2	92.6	7.7

^a^ EBOV/Makona isolate was used for the virus inoculum.

^b^ Endpoint of the EBOV fluorescent drug screen assay.

^c^ The percentage of EBOV-positive cells was determined with a VP40/Alexa-594 antibody stain to detect EBOV and a nuclear stain to quantify the number of cells in a high-content imaging system.

^d^ S/N is the ratio of the fluorescent signal (mean from 3 infected wells) to the noise (mean from 3 mock-infected wells) quantified using a plate reader.

Abbreviations: EBOV^+^, Ebola virus-positive; hpi, hours post-inoculation; MOI, multiplicity of infection; S/N, signal-to-noise ratio.

**Table 2:** Effect of time postinoculation and virus inoculum on the fluorescent EBOV drug screen assay on Huh 7 cells.

MOI^a^	24 hpi^b^		48 hpi		72 hpi		96 hpi	
	EBOV^+^ (%)^c^	S/N^d^	EBOV^+^ (%)	S/N	EBOV^+^ (%)	S/N	EBOV^+^ (%)	S/N
**0.0010**	0.1	1.2	0.0	0.9	0.1	1.1	3.0	1.0
**0.0033**	0.1	1.1	0.6	1.8	0.1	1.3	0.3	1.1
**0.01**	0.0	0.7	0.0	1.1	2.1	1.5	3.3	2.7
**0.03**	0.0	1.1	0.1	1.2	2.7	1.8	24.8	3.4
**0.10**	0.0	0.9	4.9	0.9	33.0	6.9	82.4	7.8
**0.33**	0.3	0.9	24.1	4.3	89.0	10.1	71.9	9.1
**1.00**	2.6	2.6	70.7	6.4	82.3	7.4	61.1	9.0
**3.33**	8.4	3.4	86.1	10.3	74.0	9.4	61.8	8.4

^a^ EBOV/Makona isolate was used for the virus inoculum.

^b^ Endpoint of the EBOV fluorescent drug screen assay.

^c^ The percentage of EBOV-positive cells was determined with a VP40/Alexa-594 antibody stain to detect EBOV and a nuclear stain to quantify the number of cells in a high-content imaging system.

^d^ S/N is the ratio of the fluorescent signal (mean from 3 infected wells) to the noise (mean from 3 mock-infected wells) quantified using a plate reader.

Abbreviations: EBOV^+^, Ebola virus-positive; hpi, hours post-inoculation; MOI, multiplicity of infection; S/N, signal-to-noise ratio.

Based on the data obtained from Fig 2 and Tables 1 and 2, two optimized conditions for the fluorescence assays were selected. An MOI of 1 with 48 hpi as endpoint and an MOI of 0.1 with an endpoint of 72 hpi produced a robust signal for all 3 cell types, Vero E6, Huh 7 and MDMs (Fig 2A–2F, Tables 1 and 2). Vero E6 cells were infected with EBOV at around 17.1%, and MDMs and Huh 7 cells usually had a stronger signal with around 80% or higher of EBOV-positive cells (Fig 2C–2F). The two conditions had a similar signal or EBOV infection rate. At the lower MOI of 0.1 acceptable signal was observed at 72 hpi. If cells were inoculated with higher MOI of 1, the infection time for an acceptable signal could be reduced to 48 h.

### Effect of cell passage number on EBOV infection

**Fig 3:**
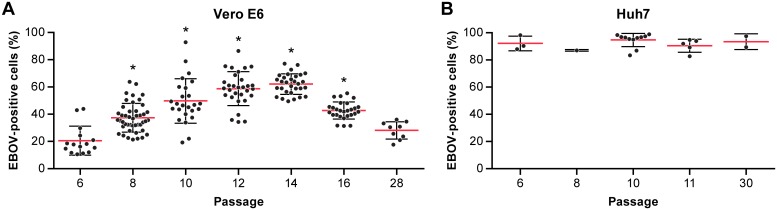
Impact of cell passaging on EBOV infection. Vero E6 (A) and Huh 7 (B) cells were passaged and infected with EBOV/Mak at an MOI of 1 at 48 hpi, the plates were fixed, stained, and the percentage of EBOV-positive cells was determined by HCI. Each point is the mean of 3 replicate wells and represents an independent experiment. For each passage, the median value of all experiments was determined. Abbreviations: EBOV, Ebola virus; HCI, high content imaging; hpi, hours post-inoculation; Mak, Makona.

During the assay development phase, Vero E6 cells appeared less reliable in producing a consistent viral spread, and this lack of reliability appeared to be due to cell culture passage history. To address this matter, Vero E6 and Huh7 cells were analyzed at different cell passage numbers by determining percentage of EBOV-positive cells (Fig 3A and 3B). Both cell types were infected with a MOI of 1 for 48 h. The viral spread of Huh 7 cells were consistent over cell culture passages 6–30, indicating that the passage number does not impact EBOV spread in Huh 7 cells in the range tested. In contrast, EBOV spread on Vero E6 cells varied considerably between cell passages 6–28 with peak infection (replication efficacy) at cell culture passages 12–14 (Fig 3A) and equally lower for early or late passages. Significant differences were observed at passages 8, 10, 12, 14, and 16 when compared to the passage 6 or 28 using an ordinary one-way ANOVA following Dunnett’s Multiple Comparison in Graphpad Prism 7.0.

**Fig 4:**
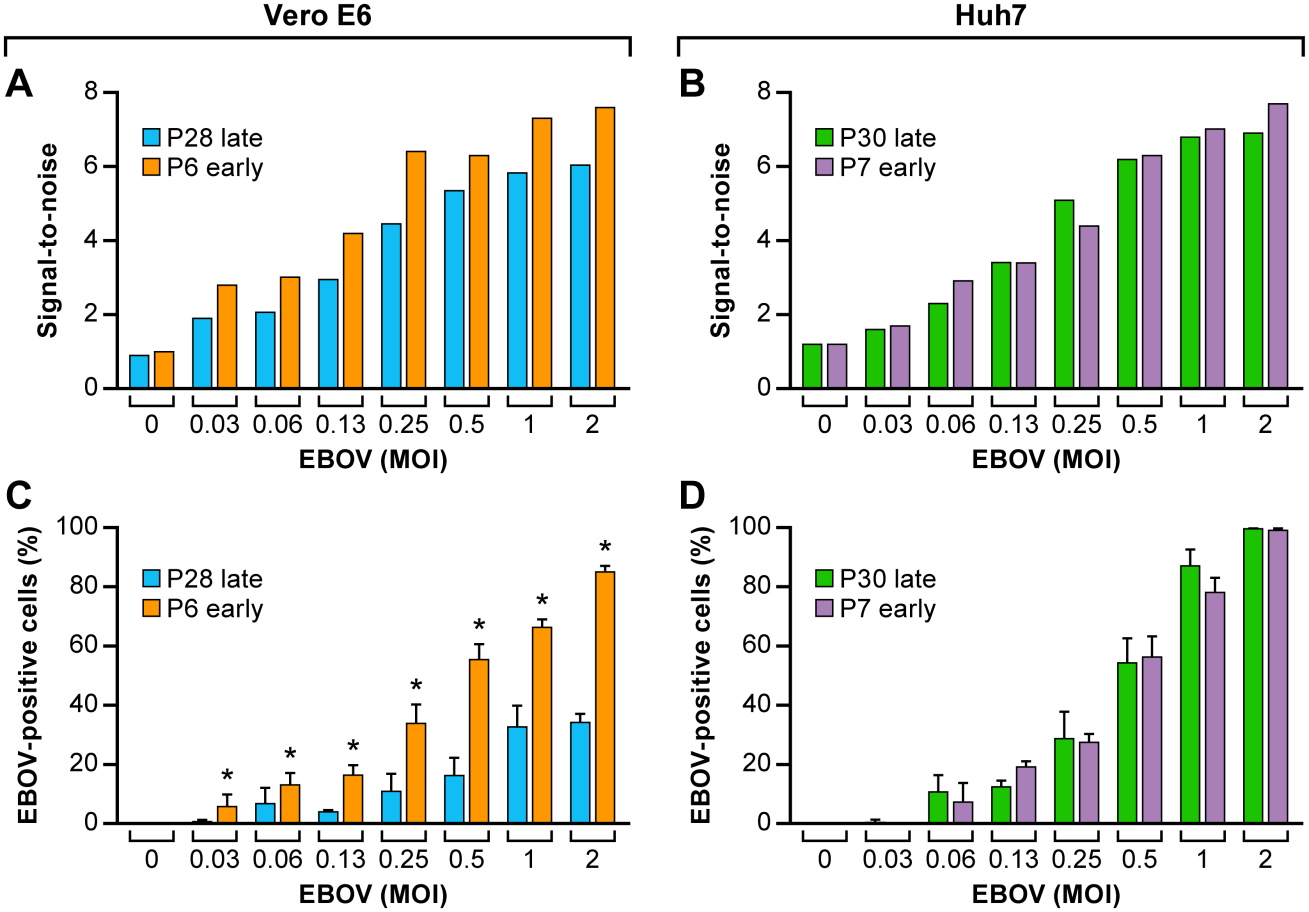
Infectivity of EBOV at an early and late cell passage of Vero E6 and Huh 7 cells. The signal-to-noise ratio (Tecan plate reader) was determined for Vero E6 (A) cells at passage 6 and 28 and for Huh 7 (B) cells at passage 7 and 30. The cells were infected at different MOIs of EBOV/Mak for 48 h, then fixed and stained. The percentage of EBOV-positive cells (C, D) by HCI was determined in parallel. The S/N ratios were determined from the mean values (± SD, n = 3) of triplicate signal and noise wells. Values for % EBOV-positive cells were determined from triplicate wells (mean ± SD, n = 3). The data were derived from one individual experiment. Two-tailed paired t test was performed to compare the values for % of EBOV-positive cells between early and late cell passage in variable MOIs, which is significantly higher in early passage of than late passage in Vero E6 cells only. Abbreviations: EBOV, Ebola virus; HCI, high content imaging; Mak, Makona; MOI, multiplicity of infection; S/N, signal-to-noise.

Occasionally, exceptions to this trend were observed (Fig 4). The infectivity of EBOV in Vero E6 and Huh 7 cells were compared at an early (P6) and a late passage number (P28) at a range of MOIs (0.03–2.0) for 48 h. In this case, Vero E6 cells tested at the later passage showed a lower infection rate (<40%) as expected, while the early passage demonstrated an unusually high infection rate (up to 80%). Infectivity in Huh 7 cells remained consistent regardless of passage number, Vero E6 cells were inconsistent overall despite the trend originally observed.

### Factors that impact the EC_50_ and drug activity

**Fig 5:**
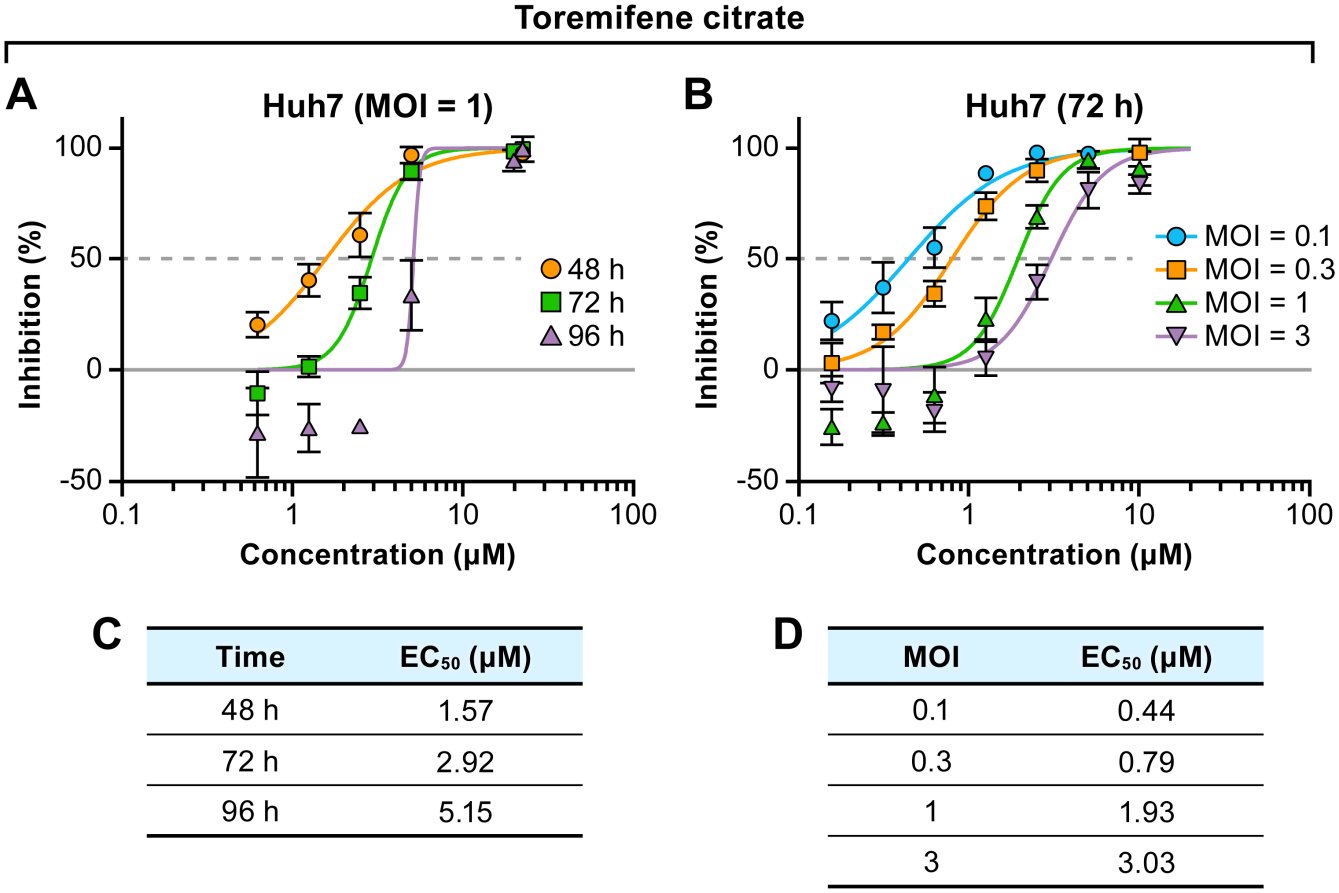
Impact of exposure time and virus input on efficacy of toremifene citrate. (A) Huh 7 cells were infected at an MOI of 1, and antiviral activity of toremifene citrate was evaluated at indicated time points. (B) Huh 7 cells were infected at indicated MOIs and antiviral activity of toremifene citrate was evaluated at 72 hpi. (C, D) EC_50_s with corresponding assay endpoints or MOIs are shown for comparison. The experiment was performed twice using the fluorescent assay. Representative graphs are shown. Abbreviations:EC_50_, half maximal effective concentration; hpi, hours post-inoculation; MOI, multiplicity of infection.

Both the virus input and duration of the experiment can have an impact on drug activity. To address this, we evaluated the *in vitro* efficacy of toremifene citrate, an FDA-approved drug with proven anti-EBOV activity [33], under different parameters using fluorescence as the read out. Huh 7 cells were infected with a constant MOI of 1 and treated with toremifene citrate for 48, 72, or 96 h (Fig 5A). Later time points resulted in a decrease in activity with the EC_50_ at 96 hpi 3.3-fold higher than at 48 hpi. When the time point remained constant at 72 hpi the activity of toremifene citrate increased as the virus input decreased (MOIs of 0.1, 0.3, 1, or 3) (Fig 5B). The EC_50_ at a MOI of 0.1 was 6.9-fold lower than at a MOI of 3.

**Fig 6:**
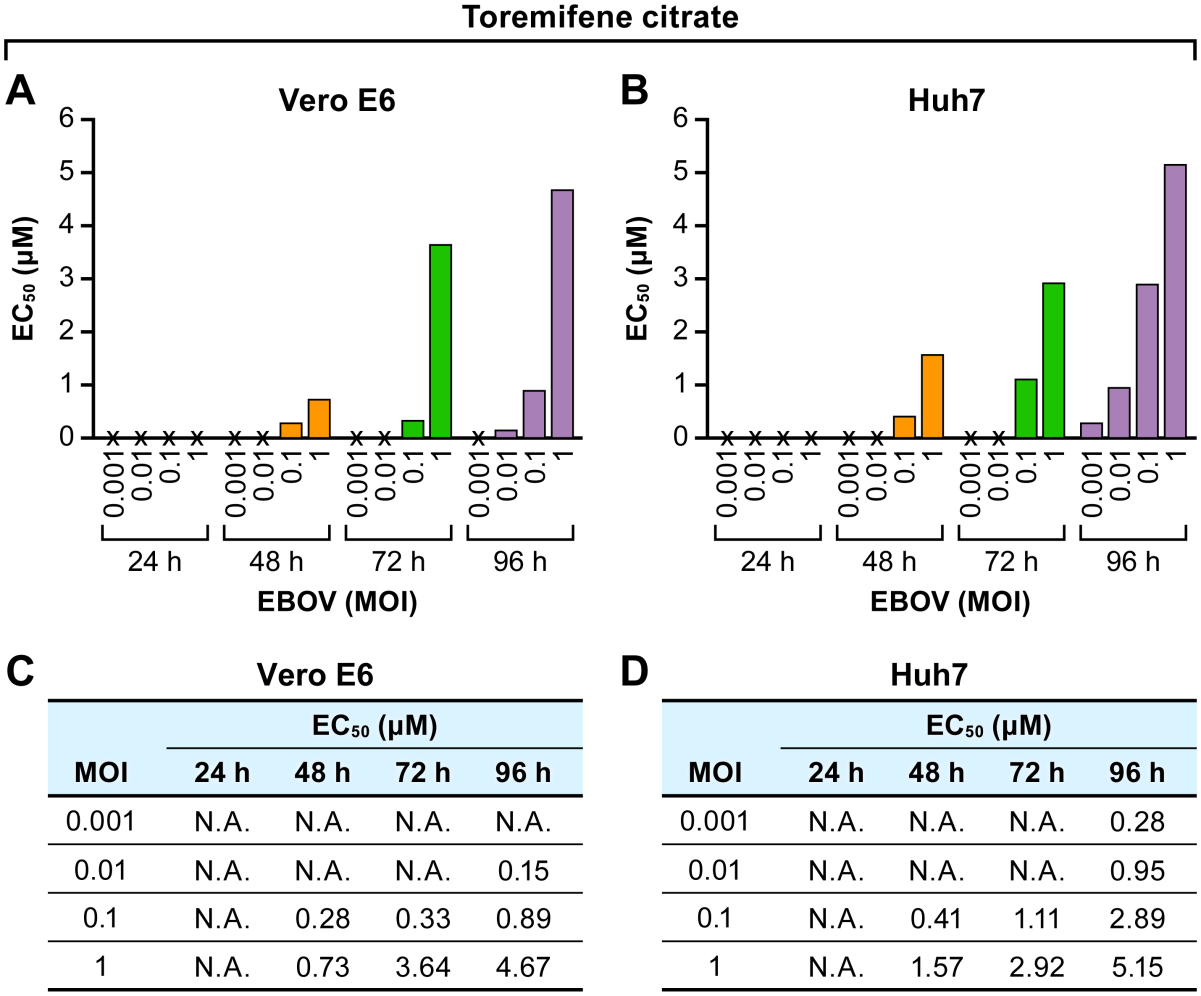
Anti-EBOV activity of toremifene citrate in Vero E6 and Huh 7 cells under different conditions. (A) Vero E6 cells and (B) Huh 7 cells were infected at varying MOIs with different assay end points and treated with toremifene citrate. EC_50_s were determined from 8-point dose response curves using the fluorescent assay. (C, D) EC_50_s of toremifene citrate with corresponding assay end points or MOIs are shown for comparison. Representative graphs from 1 to 4 independent experiments are shown. Abbreviations: EBOV, Ebola virus; EC_50_, half maximal effective concentration; h, hour; MOI, multiplicity of infection; N.A., not applicable.

In addition to time and virus input, the cell type used in the assay can result in differences in the calculated EC_50_. Anti-EBOV activity of toremifene citrate was measured using variable assay endpoints (48, 72 and 96 hpi), MOIs (0.001, 0.01, 0.1, and 1), and cell lines (Vero E6 and Huh 7 cells, Fig 6). EC_50_ values for toremifene citrate increased with exposure time and virus input in both cell types. Overall, activity in Vero E6 cells was higher with maximum activity (EC_50_ = 0.15 μM) at 96 hpi and an MOI of 0.01. In Huh 7 cells, maximum activity (EC_50_ = 0.28 μM) was detected at 96 hpi and an MOI of 0.001. EC_50_ values for toremifene citrate increased with exposure time and virus input in both cell types indicating that more drug is required to produce the same anti-EBOV effect.

### Chemiluminescent detection is more sensitive than standard fluorescent detection

To increase sensitivity of the drug screen assays, a chemiluminescent enzyme-linked immunosorbent assay (CELIA) was developed for evaluating compounds for anti-EBOV activity. The CELIA combines the advantage of specificity of an immunoassay with the high sensitivity of a chemiluminescent enzyme detection assay and is a simple and low cost screening assay [34, 35]. The CELIA was compared to the fluorescent assay (detected by regular plate reader or the HCI system) by testing the efficacy of toremifene citrate against EBOV infection in Huh 7 cells with a range of MOIs (0.1, 0.3, 1, and 3) at three different time points (24, 48 and 72 hpi). Assay parameters, signal-to-noise (S/N), and Z' factor, were compared between the assays (Table 3). At the earliest time point (24 hpi), the CELIA had higher sensitivity than the fluorescent detection assay as the Z' were in the acceptable range (>0.2) even at the lowest MOI of 0.1. In contrast, the fluorescent assay required higher MOIs for reliable data sets (Z'>0.2). At the 48 hpi time point, both detection methods generated high quality data sets with the CELIA providing improved Z' factors (>0.8). At 72 hpi, the CELIA and the fluorescent assay also produced excellent data using HCI for detection, while the fluorescent data detection by regular plate reader showed higher variability, maybe due to cytopathic effects. The EC_50_s and EC_90_s were determined on those data sets with acceptable Z’ (>0.2) and overall there was good correlation between the different detection methods (Table 4). All detection assays demonstrated that higher MOI and longer times of exposure led to an increase of the EC_50_ values for toremifene citrate. In contrast, the EC_90_ values showed considerably lower fluctuations under the different conditions tested.

**Table 3:** Effect of virus input and assay endpoint on the performance of EBOV drug screen assays.

Assay endpoint	MOI	Signal-to-noise ratio^a^		Z' factor		
		Fluorescence^b^	Chemiluminescence^c^	Fluorescence^c^		Chemiluminescence^c^
		Plate reader	Plate reader	Plate reader	HCI	Plate reader
**24 hpi**	**3**	22.5	348.45	0.58	0.83	0.44
	**1**	13.5	462.36	*	0.83	0.66
	**0.3**	11.5	226.31	*	*	0.8
	**0.1**	3.5	123.73	*	*	0.5
**48 hpi**	**3**	11.86	169.36	0.5	0.94	0.77
	**1**	24.96	246.02	0.43	0.94	0.86
	**0.3**	12.07	498.61	0.77	0.91	0.84
	**0.1**	15.93	533.66	*	0.84	0.7
**72 hpi**	**3**	16.05	129.64	0.29	0.82	0.92
	**1**	19.93	169.36	0.41	0.87	0.77
	**0.3**	17.7	424.49	*	0.87	0.89
	**0.1**	17.97	498.61	0.2	0.72	0.84

^a^ Signal-to-noise ratio was determined using a Tecan plate reader. The signal is the mean value from 3 infected wells, the noise as the mean value from 3 mock-infected wells.

^b^ The fluorescent drug screen assay was performed with the VP40 and Alexa-594 antibodies.

^c^ The chemiluminescent EBOV drug screen assay (CELIA) was performed with the VP40 and HRP-antibodies.

Abbreviations: EBOV, Ebola virus; hpi, hours post-inoculation; HCI, high content imaging; MOI, multiplicity of infection.

**Table 4:** Comparison of anti-EBOV efficacy of toremifene citrate under different assay conditions.

Assay endpoint	MOI	EC_50_ (μM)^a^			EC_90_ (μM)^a^		
		Fluorescence^b^		Chemiluminescence^c^	Fluorescence^b^		Chemiluminescence^c^
		Plate reader	HCI	Plate reader	Plate reader	HCI	Plate reader
**24 h**	**3**	1.38	0.92 ± 0.17	0.71 ± 0.0	14.29	4.01 ± 0.0	4.06 ± 0.40
	**1**	*	1.21 ± 0.59	0.27 ± 0.05	*	3.65 ± 0.82	5.28 ± 1.70
	**0.3**	*	0.46 ± 0.03	0.39 ± 0.0	*	2.69 ± 0.0	3.95 ± 0.46
	**0.1**	*	*	0.25 ± 0.04	*	3.27 ± 1.0	*
**48 h**	**3**	3.43 ± 0.12	3.01 ± 0.49	2.43 ± 0.14	11.14 ± 1.92	5.80 ± 0.28	5.59 ± 0.08
	**1**	1.99 ± 0.02	2.03 ± 0.20	1.37 ± 0.09	10.59 ± 0.68	5.14 ± 0.04	4.89 ± 0.07
	**0.3**	1.34 ± 0.39	0.90 ± 0.19	1.10 ± 0.71	5.18 ± 1.31	3.66 ± 0.70	3.27 ± 0.60
	**0.1**	*	0.34 ± 0.10	0.53 ± 0.08	3.82	3.14 ± 0.58	2.83 ± 0.23
**72 h**	**3**	5.22 ± 0.91	4.07 ± 0.19	3.30 ± 0.14	14.70 ± 2.51	7.44 ± 0.49	6.34 ± 0.07
	**1**	4.03 ± 0.23	2.67 ± 0.16	2.67 ± 0.10	10.19 ± 3.24	6.10 ± 0.01	5.07 ± 0.74
	**0.3**	*	2.46 ± 0.22	1.85 ± 0.29	7.76	5.54 ± 0.65	5.20 ± 0.31
	**0.1**	2.15 ± 0.51	1.03 ± 0.78	1.38 ± 0.04	6.04 ± 1.70	4.02 ± 0.33	2.90 ± 0.30

^a^ EC_50_ and EC_90_ values are mean values ± standard deviation from 1–3 dose response curves.

^b^ The fluorescent drug screen assay was performed with the Alexa-594 antibody. Fluorescence was determined with a Tecan plate reader, the percentage of EBOV-positive cells was determined by HCI.

^c^ The chemiluminescent drug screen assay (CELIA) was performed with the HRP-antibody using a Tecan plate reader.

Abbreviations: EC_50_, 50% inhibitory concentration; EC_90_, 90% inhibitory concentration; h, hour; HCI, high content imaging; MOI, multiplicity of infection.

### Activity of toremifene citrate in the CELIA

**Fig 7:**
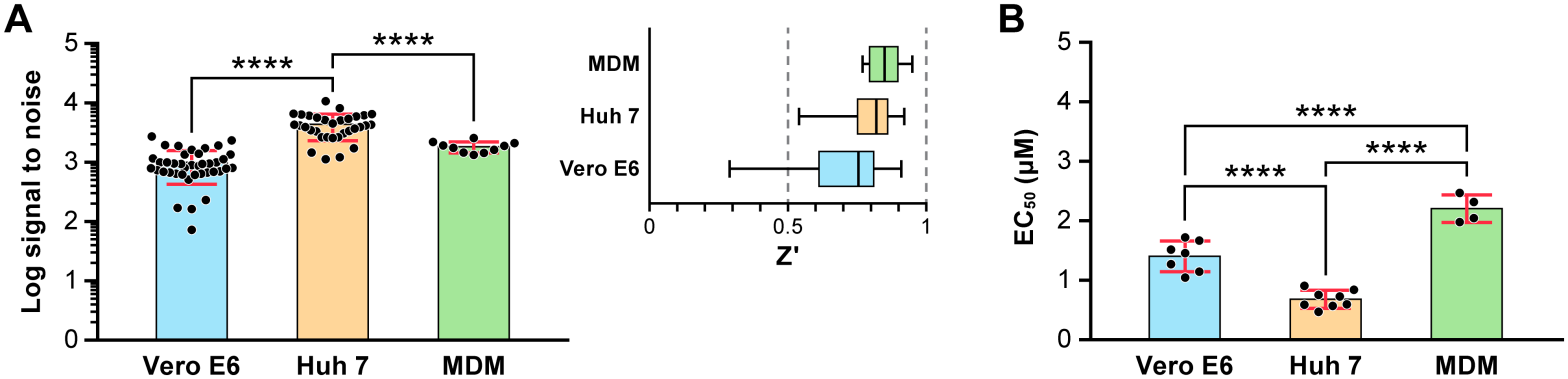
Assay parameters and toremifene activity in the CELIA. The antiviral activity of toremifene citrate was evaluated at an MOI of 0.5 (EBOV/Mak) with a 48 hpi endpoint in Vero E6, Huh 7 and MDM cells using the CELIA. (A) The signal-to-noise ratio and Z' factor were determined in multiple independent experiments. (B) EC_50_s were determined. Data were collected from 4 to over 10 experiments and the median value of all experiments was determined. Ordinary one-way ANOVA following Turkey’s post Multiple Comparison in Graphpad Prism 7.0 were performed to compare the differences of signal-to-noise ratio, Z' factor and EC _50S_ among three cell lines. Abbreviation: EBOV, Ebola virus; EC_50_, half maximal effective concentration; Mak, Makona; MDM, monocyte-derived macrophages.

The established CELIA was used to compare anti-EBOV activity of toremifene citrate in 3 different cell types, Vero E6, Huh 7 and MDMs using an MOI of 0.5 and a time point of 48 h (Fig 7). Huh 7 cells and MDMs had S/N ratios in the range of 1000s with Z' factors generally above 0.5 (Fig 7A). The S/N for Vero E6 cells ranged from >100 to over 1000, leading to more variability as reflected in the wider range of Z' factors (0.3–0.9) (Fig 7A). The activity (EC_50_) of toremifene was compared, and considerable differences were detected for the 3 cell types (Fig 7B). The efficacy of toremifene citrate was 3-fold higher in Huh 7 cells than in MDMs, and 2-fold higher than in Vero E6 cells (Fig 7B). In summary, the chemiluminescent drug screen assay for evaluating anti-EBOV activity of compounds is a very robust, reliable and reproducible method.

## Discussion

The data presented in this study exemplify the importance of having guidelines for testing drugs *in vitro* for antiviral activity and generating reproducible data sets that can be shared and confirmed by outside laboratories. Several parameters should be considered when testing drugs *in vitro* for antiviral efficacy. Cell type, assay endpoint, and the virus input are among the most important factors [36].

The Vero E6 cell line, derived by immortalization of African Green monkey kidney cells, is the most commonly used cell line for testing antivirals against filoviruses. EBOV propagates very well in this cell line, and many laboratories, including ours, generate their virus stocks and determine virus titers in Vero E6 cells. However, the argument to use a cell line with more relevance to human disease has come up repeatedly. Hence, the human liver cancer cell line, Huh 7 and human MDMs were chosen for these studies. Macrophages are relevant to human disease, and they are considered to play an important role in virus dissemination in pathogenesis of EVD [37]. Therefore, a drug that is highly effective at inhibiting EBOV infection in MDMs may better translate to *in vivo* potency than Vero E6 cells.

The susceptibility of three cell types was compared over a range of virus input and over time. All cell types were permissive to EBOV infection, but to different degrees. MDMs demonstrated optimal replication starting as early as 24 hpi at an of MOI 0.1. In contrast, EBOV grew slower in Vero E6 and Huh 7 cells. The optimal conditions for evaluation of drug efficacy using the fluorescent assay were at an MOI of 0.1 for the 72 h time point or at an MOI of 1 for the 48 h time point for all 3 cell types to ensure a strong virus signal and avoid destruction of cell layer (Fig 2G). However, an MOI of 1 is not suitable for detecting inhibitors of later steps in the virus life-cycle. The ability to detect inhibitors of virus assembly or egress will be reduced with increasing MOI, as higher proportions of the cells will be infected even in the presence of drug. For this reason, it would be advisable to use lower MOI’s, so that inhibition of virus spread within the culture can be detected.

Though Vero E6 cells are one of the most broadly used cell lines for testing compounds in *in vitro* assays, our data show that the performance of these cells is not ideal especially when evaluating drugs for anti-EBOV activity. Although it is never a good idea to passage cells for too long [38, 39], we found that at lower or higher passages the Vero E6 cells can show a decreased percentage of EBOV-positive cells. Huh 7 cells showed more efficient and more reliable virus replication irrespective of passage number. Immortalized cancer cell lines are in general an easy choice for *in vitro* drug testing because they are easy to handle, propagate, expand, and plate on a week-by-week basis with relative consistency. In contrast, primary cell types such as human primary macrophages do not proliferate in cell culture and are ideally generated fresh from human blood upon use. For large scale testing, procuring the blood volumes needed for generating MDMs from a cumbersome isolation technique could be a challenge. MDMs demonstrated very efficient spread of virus through cell culture over time. Donor-to-donor variability precludes consistency, and in general, macrophages from different donors are not pooled. Despite of the constraints, testing a selection of drugs that are closer to human clinical studies in human macrophages makes sense to get a better idea on efficacy in a more relevant target cell.

Fluorescence was detected by two different read outs and each technique has its advantages. The plate reader will measure the average fluorescence across the whole well. Readings are quick and conducive to handling large numbers of plates during screening. However, the quality of the cell layer cannot be monitored. HCI provides images of 1 or more fields of each well, and a nuclear stain can be added in parallel to indicate the viability of the cell layer. In the assay development phase, this tool is especially useful in identifying conditions that will ensure a healthy cell layer or identify issues such as plate corner or edge effects for assay quality control. One drawback is that not the whole well is imaged, but only a certain number of fields inside the well. It is important to pick the fields wisely to avoid bias or skewing of data. Scanning at least 8 fields per well is recommended for statistical purposes, but that will increase reading time per plate. The time to read and analyze the HCI data can take considerably longer than the data acquired with the regular plate reader. HCI measures different parameters such as percentage of EBOV-positive cells and mean signal intensity per cell. Both the regular plate reader and HCI have unique applications in a drug testing program. While a plate reader is good for a quick readout of large number of plates, the HCI system is used to cross check on quality of cells and to clarify issues of noise and signal variability.

In addition to the fluorescent assay, we also implemented a CELIA using an HRP-labeled antibody, which amplified the signal and increased the sensitivity of virus detection. As expected, the CELIA showed an improvement in the quality of data sets compared to the fluorescent assay. S/N ratio and Z' factor were in an acceptable range as early as 24 hpi with the lowest virus input detected at an MOI of 0.1. An EC_50_ could also be determined at this time point. At 48 or 72 hpi, the two fluorescent read outs were similar to the chemiluminescent read out.

Toremifene citrate is a selective estrogen receptor modulator reported to be active *in vivo* against EBOV in mice [12, 33]. This is an FDA-approved drug for treating breast cancer [12]. Mechanism of action studies showed that toremifene citrate affects EBOV virus entry at the stage of virus-fusion with the endosomal membrane [40]. Toremifene citrate was chosen by the WHO as a potential candidate for clinical evaluation in EVD patients. We evaluated the performance of toremifene citrate on anti-EBOV activity using different conditions.

A trend towards higher EC_50_s with increasing input virus and longer assay time was observed. The data indicate that if the amount of virus present in cells or tissues is high enough, the drug will be less active. Also with longer time for the virus to replicate, drugs may be unable to stem high viral replication. In contrast, the EC_90_ values were less affected by high MOI or longer time points, which may, therefore, be a more reliable parameter for comparing data sets between laboratories.

Serum in the media and pretreatment of cells with the compound can also have a considerable effect on antiviral activity. Toremifene was compared directly to brincidofovir under a panel of different conditions [41] showing that activity of brincidofovir was dependent on media conditions with low serum (< 5%) and 48 h pretreatment before adding virus. In contrast, the serum concentration had no effect on the activity of toremifene citrate, and pretreating cells for 48 h (instead of 1 h) decreased the activity of this drug.

Many reports on the repurposing of FDA-approved drugs discuss and compare a drug’s *in vitro* EC_50_ (determined in cell cultures) with its maximum concentration in human plasma (determined in clinical trials) when evaluating the potential of the drug to have *in vivo* activity. However, our data show that the EC_50_ of a drug can range over more than a log based on testing conditions (e.g, MOI, time of endpoint, serum). Johanson et. al.[12] reported EC_50_s of 1.73 and 0.97 μM for toremifene with two different EBOV strains EBOV/Kik and EBOV/May, respectively, at 48 hpi with 0.4 MOI by CELIA using the 13C6 antibody. These data are comparable to the EC_50_ of 1.10 ± 0.71 μM for EBOV/Mak determined in our assay under similar conditions (Table 4; MOI 0.3/ 48 hpi). Understanding how assays are performed and the potential variables is important, and caution should be used when using *in vitro* data for making decisions to advance a drug to *in vivo* studies.
